# The Mounting Evidence for Permissive Hypercreatinemia in Acute Heart Failure: Adding Sodium Glucose Cotransporter Inhibitors to the List

**DOI:** 10.34067/KID.0000000000000266

**Published:** 2023-10-26

**Authors:** Etienne Macedo, Nicholas Wettersten

**Affiliations:** 1 Division of Nephrology, University of California, San Diego, La Jolla, California; 2 Division of Cardiovascular Medicine, San Diego Veterans Affairs Medical Center, San Diego, California; 3 Division of Cardiovascular Medicine, University of California, San Diego, La Jolla, California

**Keywords:** AKI, heart failure

Sodium/glucose cotransporter 2 inhibitors (SGLT2is) have been a pharmacologic breakthrough in the management of CKD and heart failure (HF). However, with their relative newness in clinical use, these agents are frequently held because of concerns of worsening kidney function and AKI. These concerns largely stem from the occurrence of AKI during safety profile analysis of large randomized clinical trials with SGLT2is and from postmarketing reports that prompted the US Food and Drug Administration (FDA) to issue a warning that SGLT2is might cause AKI. The hemodynamic mechanism of action of SGTL2is corroborated with the concerns for AKI. By inhibiting sodium/glucose cotransporter 2 (SGLT2) in the S1 segment of the proximal convoluted tubule, where 90% of the filtered glucose reabsorption occurs, the consequent glucosuria and natriuresis increase macula densa sodium chloride delivery, stimulating tubuloglomerular feedback, resulting in afferent arteriolar vasoconstriction and reduced intraglomerular pressure. This hemodynamic effect can lower BP, induce a mild acute decline in eGFR, and raise creatinine at times enough to fulfill the creatinine-based criteria for AKI.^1^

However, despite this mechanism of action and contrary to the initial concerns, several large, prospective, and randomized studies and a meta-analysis have failed to show an increased AKI risk with SGLT2i and instead showed that they may even reduce AKI risk.^2–4^ SGTL2is may provide metabolic effects against AKI similar to the posited cardioprotective benefits. SGLT2i is believed to increase myocardial energy consumption of ketone bodies, which leads to a more energy-efficient cardiac workload. Similar findings have been reported in the kidneys where SGLT2i reduces the use of glucose and shifts metabolism to fatty acids and/or amino acids for oxidative phosphorylation. In addition, new findings have shown that increases in urine volume during SGLT2 inhibition are transient despite persistent glucosuria, suggesting osmotic diuresis to be limited with long-term SGLT2 inhibition, despite the continued excretion of glucose solutes.^5^ These hemodynamic and metabolic changes triggered by SGLT2is underlie the kidney protection.^6^

Despite the accumulating evidence over the past 5 years on the beneficial actions of SLGT2is on acute and CKD, these agents are still often withheld in patients at risk of AKI or during early stages of kidney injury. Because most kidney protective studies have been conducted in the outpatient setting, Aklilo *et al.* performed a retrospective analysis from the five hospitals in the Yale Health System aiming to gain clinical insights into the safety of continuing or starting SLGT2is in the setting of hospitalization with acute HF (AHF) in patients who develop AKI. The authors assessed the effect of SGLT2is on 30-day mortality and rate of renal recovery among AHF patients with AKI exposed and unexposed to SGLT2is within the first 10 days of AKI diagnosis. To adjust for potential biases introduced by the differences from nonrandom allocation of treatments and the potential effect of immortal bias because of different timing of SGLT2i initiation, the authors applied a sophisticated statistical analysis plan. First, they calculated a propensity score representing the probability of receiving SGLT2is using a logistic regression model that incorporated 36 covariates. Different than a traditional propensity score, they applied the inverse of the propensity score or the inverse probability of exposure for adjusting the risk in the exposed group and the inverse of the complementary probability (1—propensity score) for the non-exposed group. This strategy improves the balance of the covariates across treatment groups, making the groups more likely comparable and reducing the effect of confounding by covariates. In addition, to account for differences in duration of exposure to SGLT2i, the authors modeled SGLT2i as a time-varying covariate in the Cox regression analysis, so exposed individuals contributing time to their cohort only after SGLT2i receipt.

Using this approach and a definition for kidney recovery of a decline in serum creatinine (sCr) to a level below the threshold for the sCr Kidney Disease Improving Global Outcomes AKI criterion, they found the rate of kidney recovery over time was not proportional between the groups, but there was no difference in time to recovery or rate of recovery at 14 days in the SGLT2i-exposed and unexposed groups. Similar results were found after modeling with a term for interaction with time to address the bias resulting from the violation of the proportional hazards assumption. Time to recover was also similar between groups. In addition, the authors found that individuals prescribed SGLT2is had a remarkable 55% lower risk of death at 30 days. Overall, these findings make a compelling argument for the continuation of SGLT2is in AKI.

However, there are some important factors to consider when interpreting and applying these findings. First, the cause of AKI may be distinctly different in the exposed versus unexposed group, and AKI in AHF is not the same as AKI in other conditions, such as sepsis. Kidney function changes dynamically in many patients hospitalized with AHF,^7^ and stage 1 AKI is a common occurrence. In this study, stage 1 AKI was the highest stage in 69% of the unexposed group and 75% in the exposed group, and the median time to recovery was <2 days in both groups. Thus, considering both the magnitude of serum creatinine elevation and the duration of AKI, the transient mild elevation of sCr was likely a result of hemodynamic fluctuations from diuresis or medications.^8,9^ However, the unexposed groups were significantly less often on a loop diuretic before an AKI event (64% versus 80%), suggesting that the unexposed group was less likely to have hemodynamic-associated AKI and thus less prone to be affected by the hemodynamic effects of SGLT2is. In this scenario, it is also not expected that SGLT2is would further interfere with kidney recovery. However, on the basis of the data from this study, it is still unclear whether in patients with more severe kidney injury, SGLT2is can be safely continued.

Second, patients in the SGLT2i-exposed group more often had HF with a reduced ejection fraction (HFrEF) while most patients in the unexposed group had HF with a preserved ejection fraction (HFpEF). It is important to note that empaglfilozin was not FDA-approved for HFpEF until February 2022, 3 months before the end of this study period, and dapaglifozin, the main SGLT2i used in this study, was not FDA-approved until May 2023. While probably used off-label before FDA approval, as the authors noted cardiologists in the Yale system were allowed to use SGLT2i as adjuvant diuretics in AHF, SGLT2i was likely not applied as readily in patients with HFpEF than in patients with HFrEF before 2022. Furthermore, the primary benefit of SGLT2is in patients with HFpEF is a reduction in HF readmission and not mortality^10^; thus, even if patients with HFpEF in the unexposed group were given a SGLT2i, it is unlikely they would derive mortality benefit. Mortality rates are similar in patients with HFpEF and HFrEF; however, patients with HFpEF more often die of non-HF and non-cardiac causes of death, which again is a risk SGLT2i is less likely to mitigate.

While these are important considerations when translating these findings into real-world practice, we agree that continuing SGLT2i in patients with mild AKI is warranted, especially given that when these drugs are discontinued during AKI and are frequently not resumed at discharge. However, it may be important to differentiate between initiation and continuation because the initial hemodynamic effect can worsen kidney perfusion acutely and may delay AKI recovery in more severe injuries. In addition, these findings reassert the importance of continuing guideline-directed medical therapy in patients with AHF regardless of kidney function. This is especially important so as not to commit the same errors from the past. Previously, *β*-blockers and angiotensin antagonists were often withdrawn during episodes of AHF or AKI. Years went by though until it was recognized that this practice was unnecessary in most patients and potentially harmful. With these findings, hopefully we will not repeat the past mistakes, waiting for years to learn that holding SGLT2is leads to harm. A little permissive hypercreatinimenia in HF may go a long way.
